# Investigation of Hydrogen Sulfide Gas as a Treatment against *P. falciparum*, Murine Cerebral Malaria, and the Importance of Thiolation State in the Development of Cerebral Malaria

**DOI:** 10.1371/journal.pone.0059271

**Published:** 2013-03-26

**Authors:** Brian DellaValle, Trine Staalsoe, Jørgen Anders Lindholm Kurtzhals, Casper Hempel

**Affiliations:** 1 Centre for Medical Parasitology, Department of Clinical Microbiology, Copenhagen University Hospital, Copenhagen, Denmark; 2 Department of International Health, Immunology, and Microbiology, University of Copenhagen, Copenhagen, Denmark; Liverpool School of Tropical Medicine, United Kingdom

## Abstract

**Introduction:**

Cerebral malaria (CM) is a potentially fatal cerebrovascular disease of complex pathogenesis caused by *Plasmodium falciparum*. Hydrogen sulfide (HS) is a physiological gas, similar to nitric oxide and carbon monoxide, involved in cellular metabolism, vascular tension, inflammation, and cell death. HS treatment has shown promising results as a therapy for cardio- and neuro- pathology. This study investigates the effects of fast (NaHS) and slow (GYY4137) HS-releasing drugs on the growth and metabolism of *P. falciparum* and the development of *P. berghei* ANKA CM. Moreover, we investigate the role of free plasma thiols and cell surface thiols in the pathogenesis of CM.

**Methods:**

*P. falciparum* was cultured *in vitro* with varying doses of HS releasing drugs compared with artesunate. Growth and metabolism were quantified. C57Bl/6 mice were infected with *P. berghei* ANKA and were treated with varying doses and regimes of HS-releasing drugs. Free plasma thiols and cell surface thiols were quantified in CM mice and age-matched healthy controls.

**Results:**

HS-releasing drugs significantly and dose-dependently inhibited *P. falciparum* growth and metabolism. Treatment of CM did not affect *P. berghei* growth, or development of CM. Interestingly, CM was associated with lower free plasma thiols, reduced leukocyte+erythrocyte cell surface thiols (infection day 3), and markedly (5-fold) increased platelet cell surface thiols (infection day 7).

**Conclusions:**

HS inhibits *P. falciparum* growth and metabolism *in vitro*. Reduction in free plasma thiols, cell surface thiols and a marked increase in platelet cell surface thiols are associated with development of CM. HS drugs were not effective *in vivo* against murine CM.

## Introduction

*Plasmodium falciparum* malaria accounts for an estimated 1 million deaths annually worldwide [1]. A large portion of this burden is associated with cerebral malaria (CM), a potentially fatal cerebral complication primarily afflicting children in malaria endemic regions. CM pathology is associated with parasite-cerebrovascular interaction, platelet activation, hypoxia, inflammation, blood-brain barrier disruption, and demyelination [2], [3], [4], [5], [6], [7]. CM pathogenesis is however incompletely understood, hampering the development of effective, adjunctive therapy. Murine models for CM share several traits with human CM and have been used extensively in studies of pathogenesis and intervention [8], [9].

Hydrogen sulfide (HS) is a biologically active physiological gaseous transmitter similar to carbon monoxide (CO) and nitric oxide (NO). Indeed, much crossover in effects of these three gases has been noted including vasodilation, vascular remodeling, inhibition of apoptosis, inflammation and neuromodulation [10], [11], [12], [13], [14], [15]. Interestingly, NO and CO have been shown to be protective in murine models of CM [16], [17]. HS treatment has shown to be protective against neurodegeneration, neuroinflammation, and neuronal apoptosis and in studies of atherosclerosis, shock and also cardiac arrest [12], [14], [18], [19], [20], [21], [22], [23], [24] where a Phase II clinical trial is underway [25].

HS can thiolate proteins, reinstate depleted glutathione levels, modulate cellular and extracellular redox state, and regulate cell metabolism and cell growth [13], [26], [27], [28], [29] and thus, may be effective in controlling cellular stress and potentially parasite growth. Interestingly, two low-molecular weight thiols, pantethine and cysteamine, have been shown to reduce development of murine CM and exert partial inhibition of malaria parasite growth, respectively [30], [31].

These data on the therapeutic potential of HS for treatment of cerebrovascular disease, previous thiol-related effects on CM development and parasite growth, and the efficacy of two similar physiological gases prompted our investigation of HS as a potential treatment against *Plasmodium* proliferation and its cerebral complications, CM. In this investigation, we studied the effects of sodium hydrogen sulfide (NaHS), a fast-releasing donor of HS, and GYY4137 (GYY), a slow-releasing donor [28], [32], on the proliferation and metabolism of *P. falciparum*-infected human erythrocytes in vitro and on the development of CM in a well-described murine model relevant for human pathology.

## Methods

### *In vitro P. falciparum* Growth Inhibition and Metabolism

Stock *P. falciparum* parasites of the 3D7, PA, CSA-selected PA (PA-CSA) [33], and HB3 strains maintained in Centre for Medical Parasitology were thawed and cultured in sterile conditions at 37°C in Albumax-enriched RPMI culture media. Parasite cultivation was performed in RPMI (Gibco, DK), albumax (Gibco, DK), hypoxanthine (Sigma-Aldrich, DK), and gentamicin (Gibco, DK). Stock culture parasites were diluted to 0.5% parasitemia and cultured a volume of 1.5 mL in air-tight sterile culture flasks (TPP, 90025, Trasadingen, CH) under varying conditions. 10 ng/mL of artesunate (ART) (Sigma-Aldrich, DK) was included as a positive control for growth inhibition. Each of the four strains were cultured in saline (0.9%), ART, and increasing doses (22, 110, 550 µM) of NaHS (Sigma-Aldrich, DK). Experiments were complete after one full growth cycle of 48 hours, when parasites were enumerated [34], culture media was spun and supernatant was frozen at –80°C for metabolite analysis. Investigators were blinded to the treatment groups. Before running these experiments criteria were set to determine which strain we would continue with further investigation: a) 4% parasitemia in non-inhibited cultures after 48 hours and b) marked growth inhibition with ART. Culture strain PA-CSA met these criteria (see Results). Cultures were then repeated using this strain in five separate cultures (quintuplicate) for each increasing dose of NaHS and GYY (Cayman, Chemical, 13345-100-CAY, DK), ART, and vehicle (0.9% saline. Dosing of NaHS and GYY were calculated based on treatment doses to be used in the in vivo experiments: 0.1, 0.5, 2.5 mg/kg for NaHS and 50 mg/kg for GYY translate to doses of 22, 110, 550, 1660 µM respectively. Thus, the doses investigated were NaHS_1_ and GYY_1_∶22µM; NaHS_2_ and GYY_2_∶110µM; NaHS_3_ and GYY_3_∶550µM; representing the in vivo NaHS doses of 0.1, 0.5, 2.5 mg/kg respectively; and GYY_4_∶1660µM to translate the GYY in vivo dosing. All doses were investigated with five biological repeats (N = 5). Investigators were blinded to the treatment groups.

In a separate experiment run in parallel, culture media supernatant from uninfected erythrocytes (uRBCs) under identical conditions was assayed to determine the background metabolism of uRBCs along with infected erythrocytes (iRBCs) and culture media.

### *P. berghei* ANKA Infection and Development of Cerebral Malaria

All animal experiments were approved by the Danish Animal Experiments Inspectorate according to the license 2006/561-1128 and 2012-15-2934-00449. We aimed at group sizes of 14 mice per group for survival a study, which was powered to 80% at 95% confidence to show a 50% reduction in mortality. Technical issues resulted in lower sizes in experiment one (N = 13). Animals were kept under standard conditions with food/water access *ad libitum*, and all studies were conducted to minimize suffering and in accordance with a pre-defined humane endpoints [35]. Female C57Bl/6j (Taconic, DK) mice age 6–10 weeks were used in this study. *P. berghei* ANKA (PbA) parasites were thawed from a stock batch for which the disease course had been characterized previously (unpublished data). A pilot mouse was infected and thereafter, 10^4^ iRBCs from this mouse were used to infect mice included in each study based on previous experimental data [36]. Mice were monitored twice daily from day 4 and three times daily from onset of signs of CM. 2 µl blood samples were taken from the tail vein for parasite enumeration and 2 µl for cell surface thiol quantification as described below. CM was monitored based on presentation of ruffled fur, ataxia, hemiplegia, seizures, coma and core body temperature reduction [35], [37]. Body temperature used as an objective humane endpoint as previously described [35] with body temperature measurements below 32°C serving as an objective predictor of lethality. Terminal mice from the mid-daily interval were allocated to the late-day time interval for analysis only tested for body temperature if severe signs of CM were present. In experiment 1 and 2 body temperature was measured with a rectal probe (DM852, Ellab, DK) and experiment 3 with infrared measurement (Testo 845, Testo, Germany; [38], manuscript in preparation) and confirmed with rectal probe only when temperature was below 32°C to minimize rectal measuring. Terminal mice were anæsthetised and whole blood was collected from terminally ill mice through retro-orbital plexus puncture directly into EDTA powdered tubes (BD Biosciences, microtainer 365974, CA, US), spun and plasma was flash frozen in liquid nitrogen and stored at –80°C for plasma thiol analysis. An additional 2µl of whole blood was taken for parasitemia determination. Mice were killed by cervical dislocation and brain was observed after removal of skull for presence of hæmorrhaging to further verify the diagnosis of CM.

HS was delivered i.p. and treatment began from day 4 post infection in all three experiments to resemble a clinical case: In experiment 1, NaHS was administered once daily: Vehicle (0.9% saline), NaHS 0.1 mg/kg (N = 13), 0.5 mg/kg (N = 13), 2.5 mg/kg (N = 13). Experiment 2 was designed as a twice-daily study to increase exposure to NaHS: Vehicle (N = 14), NaHS 1.25 mg/kg (N = 14), 2.5 mg/kg (N = 15). Experiment 3 was designed to investigate a slow- HS releasing drug administered twice daily: Vehicle (N = 15), NaHS 2.5 mg/kg (N = 15), GYY 50 mg/kg (N = 15). Uninfected mice (N = 6) were included in experiment 3 in order to examine cell surface thiols of healthy, infected and drug treated mice in parallel.

### Detection of Parasitemia and Blood Metabolites

Parasites were enumerated through Giemsa-stained thin blood smears and flow cytometry using acridine orange labelling and FACScanto flow cytometer (BD Biosciences, CA, US) as previously described in detail [34].

Culture media was assayed in a blood gas and metabolite analyzer (ABL 725 analyzer, Radiometer, DK) for pH and concentration of lactate and glucose.

### Free Plasma Thiol (FPT) Quantification

FPTs were determined with thiol detection assay kit (Cayman Chemicals, 700340, DK) as per manufacturers recommendations. Briefly, samples were thawed, diluted and assessed fluorometrically according to standard curves of glutathione. 50 µl of diluted sample was mixed with 50 µl of thiol fluorometric detector provided and run in duplicate. Plates were read at excitation wavelength of 380 nm and emission wavelength 510 nm (1420 Victor Wallac multilabel reader, Perkin-Elmer, DK).

### Cell Surface Thiol (CST) Staining

2 µl blood was collected in 200 µl ice-cold heparinised PBS (50 µl/ml) and stored on ice until stained, as previously described [39]. Within two hours after blood withdrawal the suspension was centrifuged at 10 000×g for 5 minutes and the pellet was resuspended in ice-cold staining buffer (PBS, pH = 7.4, 2% fetal calf serum (Gibco, DK)). From this suspension, 10^6^ cells were used for staining. Cells were labelled for 15 minutes on ice with Alexa633-maleimide (5µM, Life Technologies, DK), a plasma membrane-impermeable dye specific to thiols. 5 µM n-ethylmaleimide (Sigma-Aldrich, DK) served as negative control. Thiol labelling specificity was controlled by preincubating cells with saturating levels of n-ethylmaleimide (0–10 µM), reducing with dithiotreitol (10 mM, Sigma-Aldrich, DK) and oxidising with hydrogen peroxide (10µM, Sigma-Aldrich, DK). The detection of thiol labelling was performed using a FACScanto flow cytometer (BD, CA, US) with a 633 nm HeNe laser and a 660/20 nm band pass emission filter (FL5) at low flow rate. A least 50,000 events were recorded for each sample. Erythrocytes and leukocytes localised closely together in forward and side scatter on the flow cytometer dot plot and were analysed together. Platelets were distinguishable on forward scatter and the identity was verified by CD41 recognition (clone Mwreg30, BD Pharmingen, CA, US). Mean fluorescence intensity (stained - negative control) was computed for each cell population. Data was analysed with FlowJo 7.6.5 (Tree Star, Inc., OR, US).

### Data Analysis

In vitro data were analysed by one-way ANOVA with Tukey’s post hoc test for multiple comparisons. Survival data were analysed with Log-Rank test. We expect a diagnosis of CM at a rate of approximately 90% in our system [36]. Thus, animals that did not develop CM based on our diagnostic measures were included in survival curves to display this incidence and excluded from further analysis. Parasitemia and weight change data were analysed with repeated measures ANOVA. FPT data between healthy and infected mice were analysed with Student’s t-test and one-way ANOVA to test differences between treatments of infected mice. For each individual mouse, CST data was normalised to baseline measurement defined as pre-infection (day 0) mean fluorescence intensity. CST data was determined to be non-parametric (q-q plot) and thereafter log-transformed. At day 3 (before treatment), a Student’s t-test compared healthy mice CST to infected mice and one-way ANOVA test compared treatment groups of infected mice from the treatment until termination. Significant differences were reported when p-value was less than 0.05. Parametric data in text is presented as mean (±95% confidence intervals) and non-parametric data with geometric mean (± standard error of geometric mean) unless otherwise stated.

## Results

### *In vitro* Metabolic and Growth Inhibition by Hydrogen Sulfide

**Figure 1:**
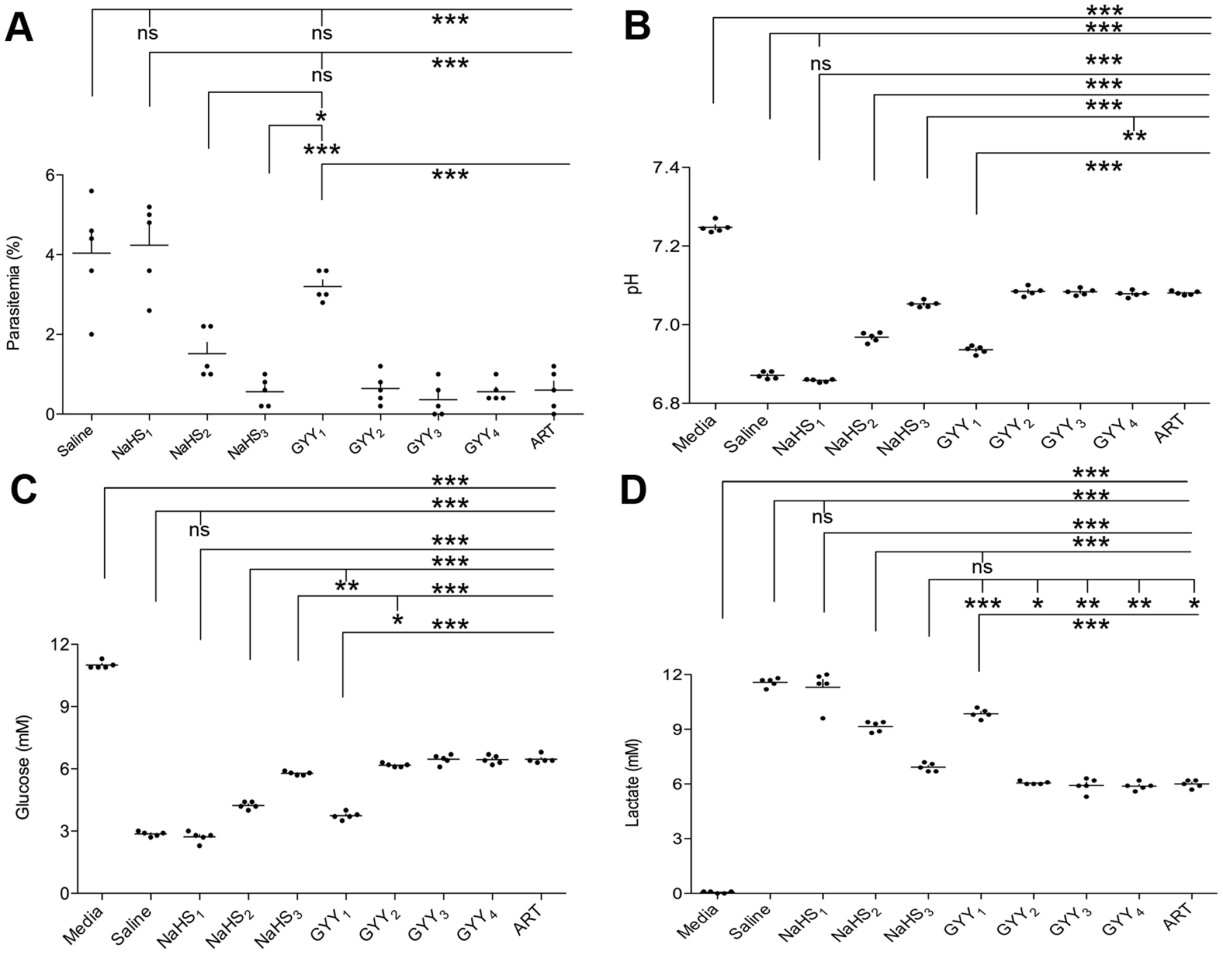
Effects of hydrogen sulfide gas on in vitro growth and metabolism of *P. falciparum*. *P. falciparum* was grown from a starting parasitemia of 0.5% in the presence of added saline (0.9%) and increasing doses of NaHS and GYY4137. After 48 hours, (a) parasitemia was enumerated and (b–d) concentration of H^+^ (pH), glucose (mM) and lactate (mM) in the culture media was quantified. Presented as dot plot of N = 5 cultures in each condition with mean (line)+S.E.M. Significant differences are denoted with asterisks (*) according to p-values of <0.05(*), <0.01 (**) and <0.001(***) as determined by one-way ANOVA with Tukey’s post hoc.

Parasitemia increased in all parasite strains after 48 hours in vehicle-treated cultures to a final level of; 3D7∶3.9%; PA: 2.6%; PA-CSA: 4.6%; HB3∶2.8%. In all *P. falciparum* strains, NaHS displayed a dose-dependent inhibition and ART fully inhibited growth in all cultures except for the HB3 culture (partial). This partial insensitivity to ART may reflect a problem with the batch and thus (in addition to 2.8% growth), was excluded from consideration for further investigation. PA-CSA was the only culture to meet the pre-defined criteria to investigate dose-response effects of HS of growth (4% infected erythrocytes) and ART inhibition. In the culturing series using PA-CSA (N = 5), vehicle-treated cultures grew to an average of 4.0 [2.3, 5.7]% parasitaemia, ART resulted in significant reduction in growth (mean: 0.6 [0, 1.2]% parasitemia) and both NaHS and GYY treatment resulted in a dose-dependent reduction in parasitaemia (Figure 1a).

In the initial experiment to define background metabolism pH, and glucose were reduced and lactate increased in cultures with cells (p<0.001 for uRBCs and iRBCs vs. media), with iRBCs showing the greatest difference irrespective of parasite strain (p<0.001 for uRBCs vs. iRBCs, Figure S1) and were similar in line with past work on *P. falciparum* [40]. In the quintuplicate series similar significant changes in pH (Figure 1b), glucose (Figure 1c), and lactate (Figure 1d) were found with the largest deviation from starting culture media levels seen in iRBC_saline_ cultures and low dose NaHS_1_. NaHS_2,3_ and all concentrations of GYY (_1–4_) reduced the changes in pH and metabolite levels (p<0.05–0.01 for treated cultures vs. iRBC_saline_).

### *In vivo* HS Treatment of CM: Parasitemia, Survival, Weight Change

**Figure 2:**
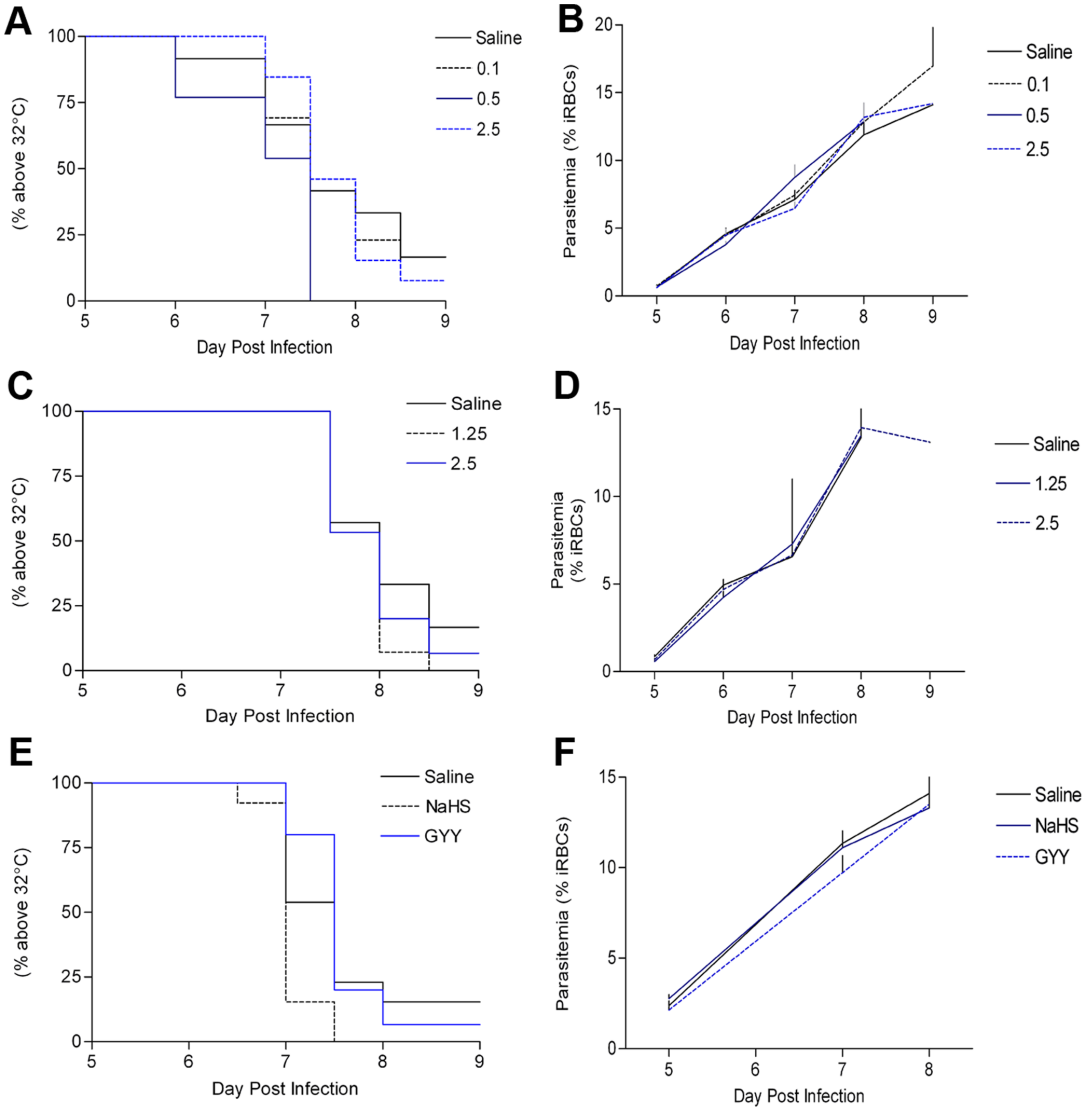
Survival analysis and parasite growth in PbA infected C57Bl/6j mice treated with HS-donating drugs. Survival data (a, c, e) and parasitemia (b, d, f) for infected mice treated with once-daily NaHS at 0.1, 0.5, 2.5 mg/kg (a,b), twice-daily NaHS at 1.25, 2.5 mg/kg, (c,d) and twice-daily NaHS 2.5, and GYY4137 50 mg/kg (e,f). All studies included a vehicle-treated (saline 0.9%) group. Survival is represented by a Kaplan-Meier plot with lethality defined as body temperature below 32°C. Parasitemia is represented as a line graph of means+S.E.M. in percentage of iRBCs in RBC population. Survival data: (a) N = 13 for all groups; (c) N = 14, 14, 15; (e) N = 15 for all groups. Parasitemia data (animals that did not develop cerebral malaria were excluded): (b) N = 11, 12, 13, 12; (d) N = 13, 14, 14; (f) N = 13, 15, 14. No significant differences were detected in the data set with Log-Rank test and repeated measures one-way ANOVA.

In experiment 1, NaHS was delivered to infected mice i.p. once daily at 0.1, 0.5 and 2.5 mg/kg along with vehicle (0.9% saline)-treated controls. The majority of mice in each treatment group ((CM_saline_: N = 11, CM_0.1_: N = 12, CM_2.5_: N = 12) with N = 13 per group) developed CM, post-mortem hæmorrhaging of the brain, and experienced body temperature reduction below 32°C. No survival differences were detected between the groups (Figure 2a). Animals that did not develop CM were included in survival analysis and excluded from remaining data analysis. Parasitemia (Figure 2b) and weight change (data not shown) did not differ between treatment groups.

Due to the short half-life of NaHS-derived HS, in experiment 2 we investigated a twice-daily i.p. treatment regime with vehicle (N = 14), NaHS_1.25_ (N = 14), and NaHS_2.5_ (N = 15). The majority of mice in each treatment group (CM_saline_: N = 12, CM_1.25_∶14 CM_2.5_∶14) developed CM, post-mortem hæmorrhaging of the brain and experienced body temperature reduction below 32°C. No survival differences were detected (Figure 2c). Parasitemia (Figure 2d) and weight change (data not shown) did not differ between groups.

Finally, we investigated a twice-daily regime with i.p. injection of a slow-releasing HS drug, GYY 4137 at a 50 mg/kg dosage (N = 15) according to previous work [21], [32] along with a vehicle treated group (N = 15) and NaHS_2.5_ group (N = 15). The majority mice in each treatment group (CM_saline_: N = 13, CM_NaHS_: N = 15, and CM_GYY_: 14) developed CM, post-mortem hæmorrhaging of the brain and experienced body temperature reduction below 32°C. No survival differences were detected (Figure 2e). Parasitemia (Figure 2f) and weight change (data not shown) did not differ between treatment groups.

### Plasma Thiol Determination

**Figure 3:**
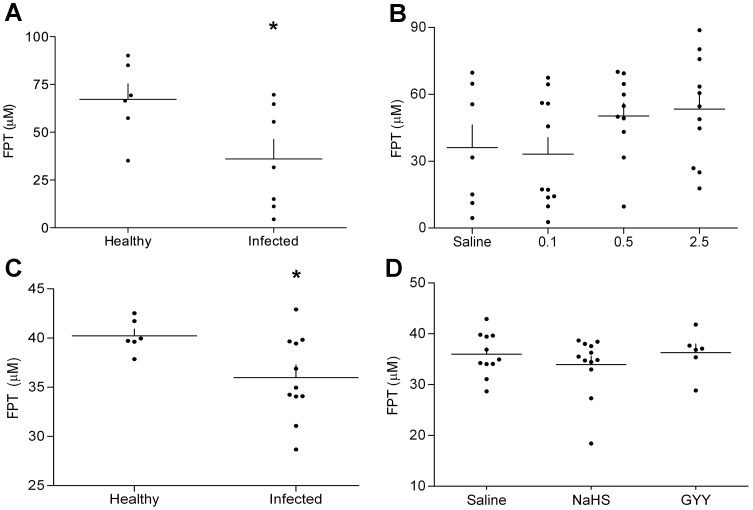
Free plasma thiol (FPT) levels in healthy and terminally ill, PbA-infected C57Bl/6j mice, and the effects of HS donors on FPTs. Plasma was collected from uninfected and terminally ill infected mice treated with saline and FPT levels were quantified in two separate experiments (a, b). Effects of HS donor drugs, NaHS and GYY4137, on FPT levels in plasma from terminally ill mice were compared to vehicle-treated terminal mice (c,d). Data is represented as dot plots with mean (line). (a) Experiment 1: N = 6, 7; Experiment 3: N = 6, 11; (b) Experiment 1∶7, 11, 10, 11; Experiment 3∶11, 12, 6. Significant differences are denoted with asterisks (*) according to p<0.05 (*) as determined by Student’s t-test and one-way ANOVA with Tukey’s post hoc.

FPT levels were determined with plasma from experiment 1 and 3 along with age-matched uninfected, healthy mice. We investigated the hypothesis that FPTs differ between healthy mice and infected mice in the terminal phase of CM. In both experiments, FPTs (values in µM) were decreased (p<0.05) in terminal CM mice (Figure 3a,c): Healthy_1_ (N = 6): 67.2 [46.4, 88.1] vs. CM_1_ (N = 7): 36.0 [11.0, 61.1] (p<0.05), Healthy_3_ (N = 6): 40.2 [38.5, 42.0], vs. CM_3_ (N = 11): 36.0 [33.2, 38.8]. We also hypothesized that HS treatment would modify the FPT levels and potentially compensate for reduced FPT sink. Treatment with NaHS or GYY in different doses did not significantly change the FPT levels (Figure 3b,d) in experiment 1 (Fig. 3b) (p = 0.2) and experiment 3 (Fig. 3d) (p = 0.5).

### Cell Surface Thiol Determination

**Figure 4:**
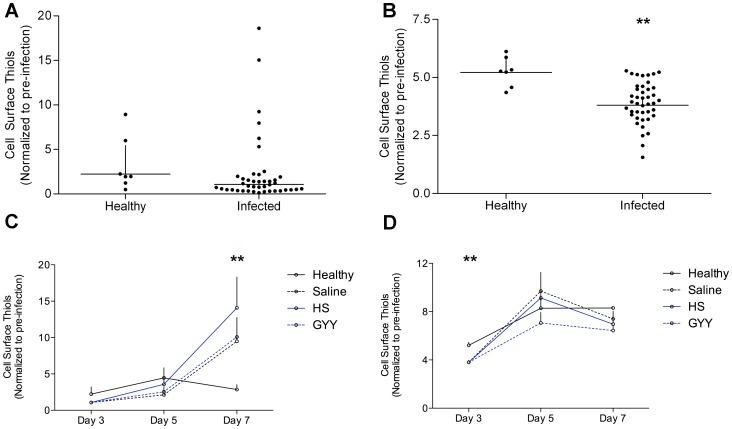
Cell surface thiol (CST) levels in healthy and PbA-infected C57Bl/6j mice, and the effects of HS donors on CSTs. CSTs of platelets (a, c) and leukocyte/erythrocyte/infected erythrocyte fraction (b, d) were analyzed with flow cytometry in healthy mice, and PbA infected mice treated with saline, NaHS (2.5 mg/kg) and GYY4137 (50 mg/kg) before infection and over the course of infection at day 3 (a, c: pre-intervention), day 5, 7 (b,d: post-intervention until terminal). All data is presented as normalized to baseline CSTs as defined by each animal’s uninfected measurements. Day 3 is presented as a dot plot (N = 7, 42) and day 3, 5, 7 are represented as line graphs of geometric mean+S.E. Geometric M (N = 7, 13, 15, 14). Significant differences are denoted with asterisks (*) according to p-values of <0.05 (*), <0.01 (**) and <0.001 (***) as determined by Student’s t-test and ANOVA with Tukey’s Multiple Comparison.

CSTs were determined in experiment 3 in treatment groups along with age-matched uninfected, healthy mice. Variation in healthy mice was observed through the course of the experiment with a slight increase up to day 5 and a reduction back to baseline by day 9 (data not shown). Due to this variation we report the mean peak fluorescence as normalized-to-baseline (day 0 before infection) for each mouse (Figure 4). At day 3 of the infection (before treatment: healthy, N = 7; infected, N = 42), CSTs on leukocytes and erythrocytes (L+RBCs) were significantly lower in infected than in uninfected mice (p = 0.003) and platelet CSTs did not differ as compared to healthy controls (p = 0.1). At day 5 post-infection (i.e. day 1 post-treatment) CSTs on L+RBCs were no longer decreased (p = 0.3) and platelets were also not different between healthy controls (N = 7) and CM_saline_ (N = 13) and between treated animals (p = 0.2). At day 7, L+RBC CSTs were not different (p = 0.3) however; platelet CSTs were markedly increased five-fold compared to healthy controls (p = 0.002). All infected mice experienced this increase and there was no difference between treated animals.

## Discussion

### Anti-parasitic Effects of Hydrogen Sulfide

In this study, we show that HS is a potent inhibitor of *P. falciparum* proliferation and metabolism in vitro. This effect of exogenous HS on *P. falciparum* has, to our knowledge, not been previously reported. The effects were dose dependent and efficient with both fast- and slow- releasing HS donors although GYY 4137 was more effective than NaHS at equal molarity. At a pH of 7.4 and 37°C, ∼20–30% of HS is in gaseous form, and thus penetration of the iRBC membrane is likely to be easily achieved [41]. Moreover, as the pH drops in the media (due primarily to iRBC metabolism (fig. 1, and fig. S1), gaseous H_2_S solubility increases. Thus, the observed effects of HS may be associated with intracellular modifications affecting the parasite directly. Indeed, efficient and reversible inhibition of mitochondrial cytochrome c oxidase in the electron transport chain by HS has been known for many years [42], [43]. These results may therefore be, in part, due to blockade of parasite mitochondrial cellular respiration, similar in class to the anti-malarial drug atovaquone [44]. Moreover, HS^−^ anions may also actively contribute to parasite stress through protein modification associated with thiolation and cellular redox balance. Elucidating the mechanism requires further investigation.

### *In vivo* Treatment of Murine Cerebral Malaria with Hydrogen Sulfide

Two other physiological gases with similar physiological effects as HS, NO and CO, have previously shown to promise as adjunctive treatment candidates for CM in murine models [16], [17]. With the overlap in actions of HS, CO, NO and the in vitro potency of HS anti-malarial activity, we hypothesized that HS may be able to concomitantly protect the brain and inhibit the parasite in vivo. Despite extensive dose testing and use of both fast- and slow-releasing HS donor molecules, murine CM did not slow in progression nor did the compounds inhibit PbA growth.

Relative to the in vitro system, HS treatment in vivo would have a much larger volume of distribution, a significantly lower half-life and acting upon a different parasite species. Given the anti-malarial potency of NaHS and GYY4137 in vitro, their low in vivo half-life, along with and the potential for HS-mediated anti-apoptotic and anti-inflammatory properties; investigations using an atmospheric HS chamber delivery system to simulate the *P. falciparum* in vitro treatment system may yield promising results. One should be aware that atmospheric HS can drastically reduce murine body temperature [29] which would negate the use of body temperature as an objective humane endpoint [35].

### *In vivo* Thiol Levels during Infection and Affects of Hydrogen Sulfide

FPTs play an important role in the maintenance of redox states important for biological interactions and the control of reactive oxygen and nitrogen species. In malaria patients, a principle de-oxifying molecule, glutathione, is decreased [45]; however, concomitantly NADPH is also reduced, precipitating an increase in cysteine-derived thiol molecules [46]. In CM mice, brain thiols are decreased with PbA infection [47]. In this study, FPTs were decreased in vehicle-treated mice. This suggests that during fulminant murine CM the body may be unable to maintain antioxidants for homeostatic redox cycling. Adjunctive treatment with the antioxidant, N-acetylcysteine in severe malaria patients did not however improve outcome [48]. In our study, HS donors did not change FPT levels compared to vehicle.

Cell surface thiols play important roles in structural integrity, biological activity, environment sensing and signaling. Indeed, it is increasingly clear that platelet surface thiols are relevant for platelet activation, secretion and adhesion [49], [50]. Importantly, platelet activation and a pro-coagulative state has been implicated as essential for development of murine CM and aggregation is prevalent in CM patients, contributing to marked thrombocytopenia [5], [30], [51]. Moreover, CSTs are important for proper lymphocyte activation and proliferation, particularly concerning proper production of cytokines [52], [53].

In this study we show that platelet surface thiols are markedly increased in fatal CM infection. A marked 5-fold increase in platelet surface thiolation was detected in infected mice when compared to healthy mice. Interestingly, this marked increase in surface thiols occurred between day 5 and 7 when the pathology becomes fulminant and approaching fatal, reflecting a marker of late-stage disease. Treatment with HS donors did not affect this increase. This CST assay should be tested along with a treatment that reverses CM to investigate the role of platelet thiolation in activation cascades and prognosis in CM.

The L+RBCs fraction experienced a reduction in surface thiols at day 3 post- infection; an effect that was absent on days 5 and 7. It has been shown that T cell CSTs are important for T cell activation and expansion of T cell subsets primarily through interleukin (IL)-2 production [52], [53]. Experimentally reduced CSTs on T cells reduces the production of IL-2 [52], [53]. Indeed, experimental potentiation of IL-2 signaling early on during infection promotes T-regulatory cell populations and protection against PbA CM [54]. This CST disturbance at day 3 (when the parasitemia is almost negligible) may play a role in the delayed immune reaction reported in mice susceptible to developing CM compared with CM resistant mice [55]. Interpretation of data after day 3 is confounded by the increasing iRBC fraction and decreasing uRBC fraction contributing to the data as a function of time. HS treatments did not affect L+RBC CSTs.

In this investigation we report a novel and potent anti-malarial effect of HS gas on *P. falciparum*. We report that *P. falciparum* is greatly inhibited metabolically and killed in the presence of HS gas from two donors, the fast-releasing NaHS and the slow-releasing GYY4137. We also report the use of a simple, robust method for testing culture media pH, glucose and lactate concentration in malaria cultures. HS treatment did not confer measurable protection against PbA proliferation and CM development in mice. We report a simple and robust method for detecting and quantifying cell surface thiols in separate leukocyte/erythrocyte/infected erythrocyte and platelet fractions with minimal blood volume during malaria infection. Finally we report that L+RBC surface thiols are reduced at an early phase of murine CM infection and in late stages, free plasma thiol levels are decreased and platelet surface thiols are markedly increased- likely contributing to the pathological condition of murine CM. Detailed investigation of the importance of platelet surface thiolation and depletion of free plasma thiols on the progression of CM are warranted and may yield important insight into CM pathogenesis and treatment.

## Supporting Information

Figure S1 **Metabolism of uninfected erythrocytes (uRBCs) and** ***P. falciparum*** **-infected erythrocytes (iRBCs) after 48 hours growth.** uRBCs and *P. falciparum* iRBCs were cultured with added saline (0.9%) for 48 hours. iRBCs were diluted to a starting parasitemia of 0.5%. Media-only wells were also included. After 48 hours, parasitemia was enumerated (not shown) and the concentration of H^+^ (pH), glucose (mM) and lactate (mM) in the culture media was quantified (a–c). Presented as dot plot of N = 4 cultures for uRBCs and iRBCs and N = 3 for media-only with mean (line)+S.E.M. Significant differences are denoted with asterisks (*) according to p-values of <0.05(*), <0.01 (**) and <0.001(***) as determined by one-way ANOVA with Tukey’s post hoc.
(TIF)
