# Supplementary figures and images for: Investigation of Hydrogen Sulfide Gas as a Treatment against P. falciparum, Murine Cerebral Malaria, and the Importance of Thiolation State in the Development of Cerebral Malaria
Source: PLoS One. 2013 Mar 26;8(3):e59271. doi: 10.1371/journal.pone.0059271 (PMC3608628; doi:10.1371/journal.pone.0059271)

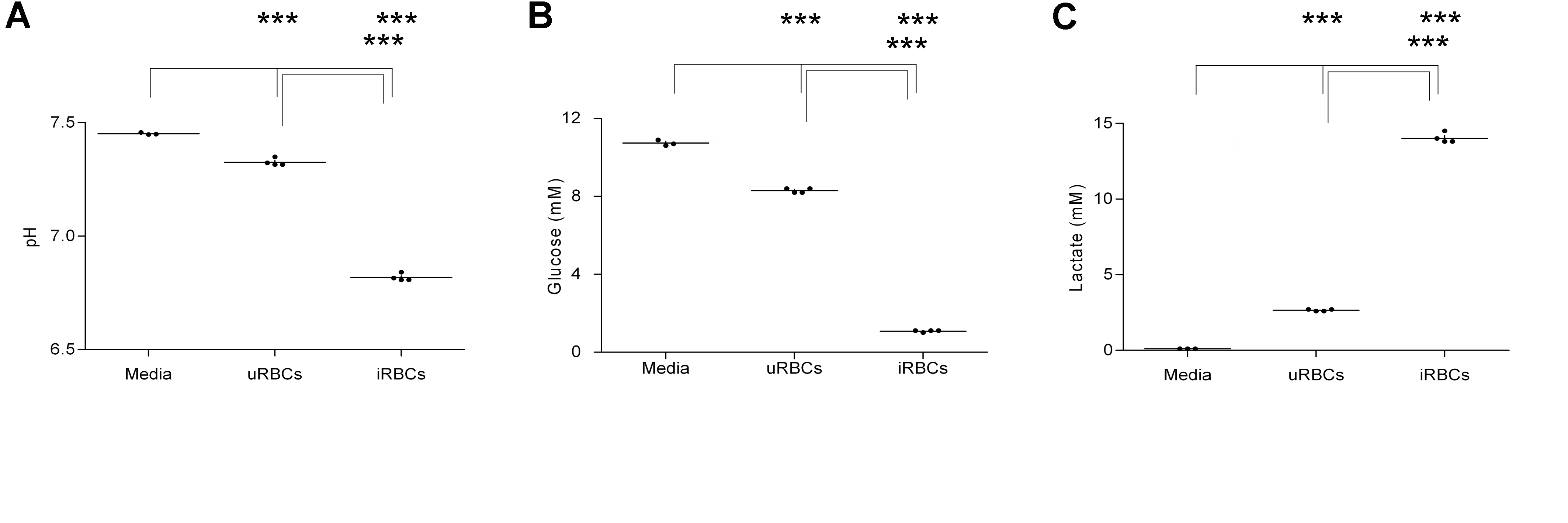

Supplement: Figure S1 — Metabolism of uninfected erythrocytes (uRBCs) and P. falciparum -infected erythrocytes (iRBCs) after 48 hours growth. uRBCs and P. falciparum iRBCs were cultured with added saline (0.9%) for 48 hours. iRBCs were diluted to a starting parasitemia of 0.5%. Media-only wells were also included. After 48 hours, parasitemia was enumerated (not shown) and the concentration of H+ (pH), glucose (mM) and lactate (mM) in the culture media was quantified (a–c). Presented as dot plot of N = 4 cultures for uRBCs and iRBCs and N = 3 for media-only with mean (line)+S.E.M. Significant differences are denoted with asterisks (*) according to p-values of <0.05(*), <0.01 (**) and <0.001(***) as determined by one-way ANOVA with Tukey’s post hoc. (TIF) [file pone.0059271.s001.tif]
